# A proposed methodology for trip recovery training without a specialized treadmill

**DOI:** 10.3389/fspor.2022.1003813

**Published:** 2022-11-21

**Authors:** Youngjae Lee, Neil B. Alexander, Michael L. Madigan

**Affiliations:** ^1^Grado Department of Industrial and Systems Engineering (0118), Virginia Tech, Blacksburg, VA, United States; ^2^Division of Geriatric and Palliative Medicine, Department of Internal Medicine, University of Michigan, Ann Arbor, MI, United States; ^3^Geriatric Research Education and Clinical Center, Veterans Affairs Ann Arbor Health Care System, Ann Arbor, MI, United States; ^4^Department of Biomedical Engineering and Mechanics (0298), Virginia Tech, Blacksburg, VA, United States

**Keywords:** trip recovery, training, falls, perturbation, balance, treadmill

## Abstract

Falls are the leading cause of accidental injuries among adults aged 65 years and older. Perturbation-based balance training is a novel exercise-based fall prevention intervention that has shown promise in reducing falls. Trip recovery training is a form of perturbation-based balance training that targets trip-induced falls. Trip recovery training typically requires the use of a specialized treadmill, the cost of which may present a barrier for use in some settings. The goal of this paper is to present a methodology for trip recovery training that does not require a specialized treadmill. A trial is planned in the near future to evaluate its effectiveness. If effective, non-treadmill trip recovery training could provide a lower cost method of perturbation-based balance training, and facilitate greater implementation outside of the research environment.

## Introduction

Falls are the leading cause of both non-fatal and fatal injuries among adults aged 65 years and older in the United States (1, 2). Falls are also costly in that the 2015 direct medical costs associated with falls among older adults in the United States totaled $50 billion (3). Falls and fall-related injuries are prevalent among older adults largely because of the declines in physical (4) and/or cognitive (5) capabilities with aging.

Trips account for 29%−53% of falls among community-dwelling older adults (6–8). These trip-induced falls frequently result from an ineffective balance recovery response to the trip-induced loss of balance (LOB) (9). Perturbation-based balance training (PBT) has received growing interest as an exercise-based fall prevention intervention (10–13), and accumulating evidence supports its ability to improve balance recovery responses as well as reduce fall rates (10). While some PBT studies have targeted disease-specific populations (14, 15), most aim to reduce falls among older adults (10, 12). The goal of PBT is to train and thus improve this recovery response. Many PBT efforts specifically target trip-induced falls (16–19). This so-called trip recovery training can improve balance recovery responses to lab-induced trips (16, 18, 20), and decrease fall rates after both lab-induced trips (16, 18) and real world trips (21). Trip recovery training has been employed using varied means to induce trips or trip-like perturbations. For example, (13, 22) used an electromechanical tripping obstacle embedded within a laboratory walkway that abruptly raised during early/mid-swing to induce a trip. Other studies have employed a specialized treadmill to elicit trip-like perturbations. For example, (16, 17) had participants stand on a stationary treadmill belt and suddenly accelerated the belt posteriorly to induce a forward LOB, while (19) had participants walk on a treadmill and applied sudden belt accelerations. Treadmill-assisted trip recovery training has been conducted using commercially-available specialized treadmills marketed for PBT (19, 23–25) as well as a lower cost option using a modified off-the-shelf treadmill (17, 20). The cost and/or space requirements associated with an electromechanical tripping obstacle within a walkway or a specialized treadmill can present a barrier to wider application trip recovery training (26). A trip recovery training regimen that does not require either may facilitate its use outside the research setting.

This paper reports a proposed methodology for trip recovery training that does not require an electromechanical trip obstacle or specialized treadmill. Successful balance recovery after a trip-induced LOB has three primary requirements: (1) quickly step anteriorly to extend the base of support and enable the ground reaction force line of action to be anterior to the whole-body center of mass; (2) quickly decelerate the forward angular velocity of the trunk segment; and (3) maintain sufficient stance limb hip height to enable stepping over the obstacle (9, 27, 28). Similar to other treadmill-based trip recovery training programs (17, 25, 29–31), the so-called non-treadmill training (NT) regimen proposed here targets these requirements through volitional step training and reactive step training, both of which can improve fall rates and risk factors for falls (32). Moreover, the step training within NT closely mimics the postures and movements required during trip recovery to leverage the specificity of training principle and thus enhance transfer to trip recovery. NT was developed by the authors based upon their expertise and experience studying trips and administering PBT among older adults. Approximately 20 pilot participants were used to refine the NT procedure described below, although no formal evaluation of its effect on trip-induced LOB responses has been completed. A trial is planned in the near future to evaluate its effectiveness on laboratory-induced trips in comparison to treadmill-based trip training and a control among community-dwelling older adults. If effective, NT could provide a lower cost method for trip recovery training and facilitate greater implementation outside of the research environment (12, 26).

## Methods

**Figure 1:**
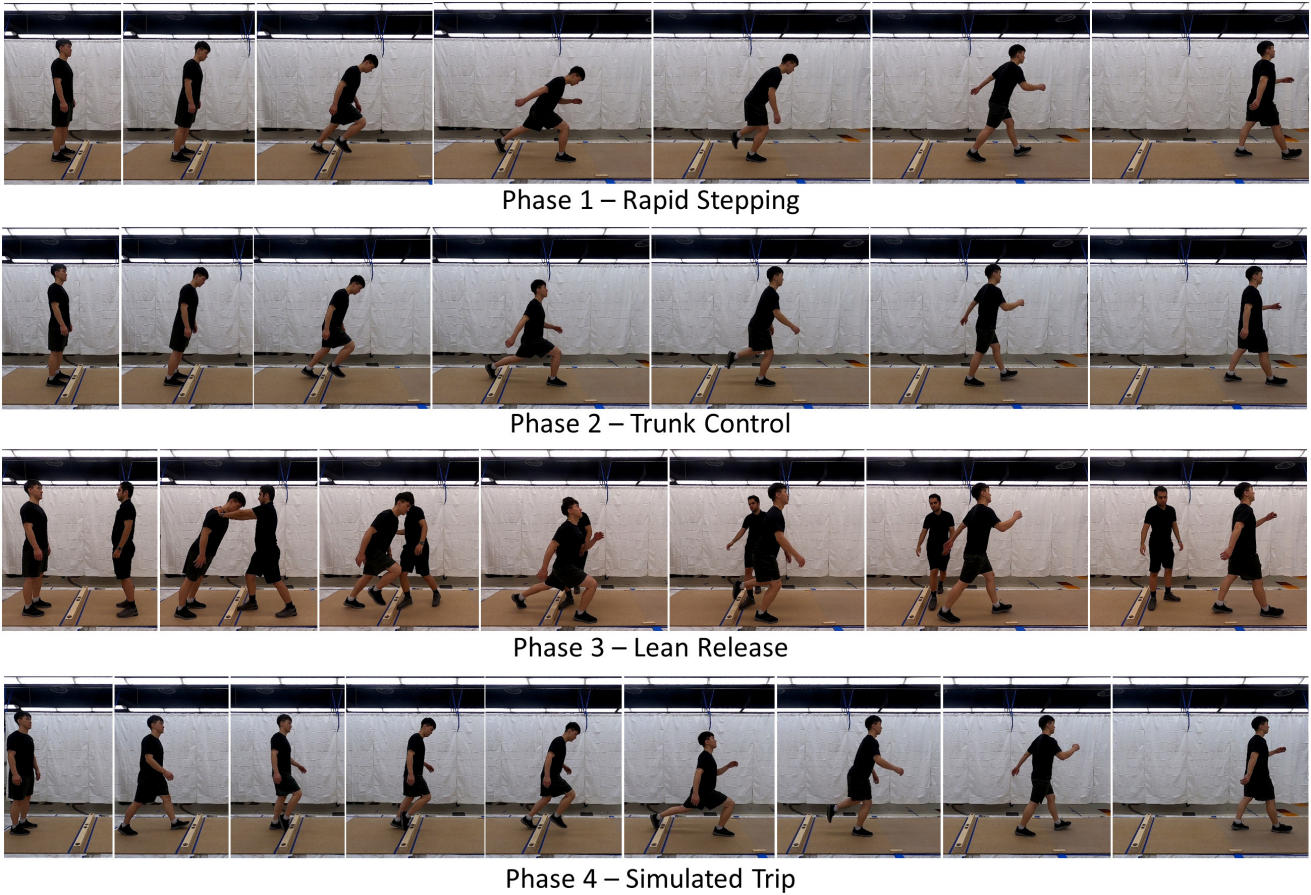
Time lapse photos of the four phases of non-treadmill training. In Phase 1, participants practiced a long and quick initial step after purposefully rotating forward about the ankles to induce a forward fall. To increase the difficulty as performance improved, participants were encouraged to delay the start of their stepping as long as possible. In Phase 2, participants also practiced a long and quick initial step after purposefully rotating forward about the ankle for as long as possible. However, emphasis was placed on controlling the sagittal plane trunk angle during the initial recovery step by aiming to achieve a vertical trunk orientation at the time of touchdown of the initial recovery step. In Phase 3, we added a reactive component by releasing participants from a static forward lean without warning. Participants focused on a long, quick initial step and trunk control as emphasized in Phases 1 and 2. In Phase 4, participants were asked to self-induced a trip while walking and practice a long, quick initial step and trunk control as emphasized in earlier Phases.

Non-treadmill training is performed over an area ~1.2 m wide by 4 m long, and uses an 8-cm-tall wooden tripping obstacle fastened to a sheet of plywood on the floor with padding affixed to vertical face where foot contact is anticipated during a trip. Each NT session includes four phases of training with increasing difficulty and similarity to actual trip recovery. Time lapse photographs of each phase are illustrated in Figure 1. Each NT session involves a single participant, is designed to be ~40 min in duration, and begins with a 3-min warm-up of walking and light stretching. The number of trials recommended within each phase is not specified because no empirical evidence is available at this time to support any such recommendation. In general, trainers should endeavor to complete a large number of trials because learning will increase with added practice, but also maintain a comfortable and enjoyable pace for the participant with time for trainer encouragement, feedback, and possible rest breaks. We anticipate multiple NT sessions being completed by participants to achieve meaningful and lasting improvements in trip recovery.

*Phase 1 – Rapid Stepping* targets the need to quickly step anteriorly to extend the base of support (27, 33, 34). It involves volitional stepping exercises from bilateral standing during which the participant starts to tip and fall forward by rotating about their ankles, and then takes quick steps to recover balance. This is repeated numerous times while the participant steps initially with the left and right feet with approximately equal frequency since trip recovery may require both. In this and all phases, participants are encouraged to complete multiple steps and achieve a stable gait even though instructions are only focused on the initial step. When the participant appears to execute these steps with little difficulty, the difficulty can be increased by encouraging the participant to fall as far forward as possible before starting to step, and also to take a long initial recovery step. Moreover, stepping is first performed without the tripping obstacle installed, and then with the tripping obstacle to elicit a step over an obstacle such as during trip recovery. The distance from the participant's initial standing position and the obstacle should initially be at a comfortable distance for stepping over (~7–20 cm), with this distance being increases as a part of making this phase more challenging.

*Phase 2 – Trunk Control* targets the need to quickly decelerate the forward angular velocity of the trunk segment (33, 35–37). It involves similar volitional stepping exercises as Phase 1, but with explicit instructions and emphasis on arresting trunk motion. To accomplish this, the participant is instructed to control their trunk segment angular orientation to be vertical at touchdown of the first recovery step. While achieving a vertical trunk segment orientation at touchdown is not a requisite for successful trip recovery, we found this to be a useful mnemonic to encourage participants to focus on controlling trunk motion. As in Phase 1, this is repeated numerous times while stepping initially with the left and right feet, initially without the tripping obstacle, and later with it.

*Phase 3 – Lean Release* targets the need to accomplish the same requirements as in Phases 1 and 2, but in response to an unexpected LOB rather than in a volitional sense. The participant leans forward while being supported bilaterally at the shoulders by a trainer standing and facing the participant with their arms fully extended. Without warning, the trainer releases the participant and steps to the side. The participant quickly takes recovery steps to recover balance. The participant is reminded to emphasize trunk segment control as in Phase 2. As in Phases 1 and 2, this is repeated numerous times while stepping initially with the left and right feet, initially without the tripping obstacle, and later with it. A verbal cue of release can be provided to the participant, if needed for confidence or frequent success. The cue can be eliminated later in training as performance improves.

*Phase 4 – Simulated Trip* attempts to integrate the requirements targeted in Phases 1 and 2 into a realistic trip. The participant starts by standing one step away from the tripping obstacle. The participant then steps with their first foot, and during the subsequent swing phase purposefully trips on the obstacle. The participant then executes an elevating strategy by using the obstructed foot to step over the obstacle, and then continues walking. As in earlier phases, this is repeated numerous times while stepping initially with the left and right feet. The participant will be instructed to emphasize taking a long initial recovery step, and controlling trunk segment by achieving a vertical angular orientation at touchdown of the first recovery step. To increase difficulty later in training, the participant can start more than one step away from the tripping obstacle.

The goal for the NT trainer should be to include all four phases during each session. However, NT can and should be individualized to each participant's capability, and completing all four phases should not come at the expense of participant comfort. Spending additional time in early phases early in the training to ensure the participant does not overexert themselves and to build the comfort and confidence with the training is likely important. Also, depending upon the physical capability of the participant and the speed at which they are able to learn the movements involved, some participants may need to spend additional time in early phases and not complete all four phases during initial NT sessions.

## Anticipated results and discussion

We anticipate NT to have a measure of acceptability among older adult participants. This expectation is based upon qualitative similarities between NT and treadmill-based trip recovery training programs and the acceptability that has been provided to the latter (26). We also anticipate NT to elicit improvements in trip recovery after laboratory-induced trips when compared to a control involving general balance and strength exercises not specific to tripping. More specifically, we anticipate improved stepping responses and trunk control following NT. This expectation is based upon a systematic review and meta-analysis indicating volitional step training and reactive step training among older adults improve fall rates and fall risk factors such as reaction time, gait, balance, and balance recovery (32). It is unclear at this time how the efficacy of NT will compare to trip recovery training using a treadmill, as well as comparing how both are received by the targeted population of community-dwelling older adults. Subsequent studies will be needed to determine how well NT transfers to fall reduction in the real-world environment.

Participants with significant lower limb joint pain gait impairment, or who are dependent upon a walking aid may not be a good fit for the proposed NT. No explicit age range is provided either given that eligibility should be based upon gait and balance ability. NT does have safety risks. As with other PBT regimens, NT risks include exacerbation of preexisting medical conditions, overexertion, tissue strains, and fall-induced injury. To minimize these risks, participants should be screened by a qualified health professional prior to NT, warmup exercises and stretching are recommended, and rest breaks can be includes as needed. The need for a safety harness is dependent upon participant physical capability and confidence level. Our upcoming trial will involve community-dwelling older adults, whom we will attempt to train with a spotter and no harness to avoid added infrastructure. NT participants should also be encouraged to wear suitable clothing and footwear.

The trainer administering NT should have requisite traits to enhance training efficacy and safety. NT as proposed here has no formal or objective quantification of participant performance during training. Because of this, modulating perturbation magnitude and difficulty so that training can be individualized and progress as the participant improves requires trainer experience and intuition. If no safety harness is used, then they should also have sufficient size and physical capacity to provide fall-arresting assistance when needed. Regardless as to whether a safety harness is used, we anticipate the trainer standing near the participant during all phases of NT to demonstrate the movements, facilitate feedback, provide physical support when needed, and also for encouragement.

We anticipate participants most likely needing to complete multiple NT sessions to achieve meaningful improvements in trip recovery. However, the number of sessions of NT needed to elicit meaningful improvements in trip recovery, as well as the optimal training schedule, have not been evaluated. We also acknowledge that only one of the four phases of the trip recovery training proposed here involves reactive stepping to perturbations that occur without warning (Phase 3). Many other trip recovery training methods reported elsewhere fully involve reactive stepping responses to sudden perturbations. While such reactive stepping appears to be more specific to balance recovery responses after perturbations, volitional stepping exercises such as those used in Phases 1, 2, and 4 can also improve fall risk factors and reduce fall rates (32), and support the potential benefits of the training proposed here.

In conclusion, a methodology for trip recovery training that does not require a specialized treadmill is presented. If acceptability by participants and effective, this training could provide a lower cost implementation of trip recovery training, and facilitate greater implementation outside of the research environment.

## Data availability statement

The original contributions presented in the study are included in the article/supplementary material, further inquiries can be directed to the corresponding author.

## Ethics statement

Written informed consent was obtained from the individual(s) for the publication of any potentially identifiable images or data included in this article.

## Author contributions

YL: conceptualization, methodology, and writing—original draft. NA: supervision and writing—review and editing. MM: conceptualization, methodology, writing—original draft, and project administration. All authors contributed to the article and approved the submitted version.

## Conflict of interest

The authors declare that the research was conducted in the absence of any commercial or financial relationships that could be construed as a potential conflict of interest.

## Publisher's note

All claims expressed in this article are solely those of the authors and do not necessarily represent those of their affiliated organizations, or those of the publisher, the editors and the reviewers. Any product that may be evaluated in this article, or claim that may be made by its manufacturer, is not guaranteed or endorsed by the publisher.
